# Acetamidine-Based iNOS Inhibitors as Molecular Tools to Counteract Inflammation in BV2 Microglial Cells

**DOI:** 10.3390/molecules25112646

**Published:** 2020-06-06

**Authors:** Silvia Grottelli, Rosa Amoroso, Lara Macchioni, Fiorella D’Onofrio, Katia Fettucciari, Ilaria Bellezza, Cristina Maccallini

**Affiliations:** 1 Department of Experimental Medicine, University of Perugia, Polo Unico Sant’Andrea delle Fratte, 06132 Perugia, Italy; silvia.grottelli@unipg.it (S.G.); lara.macchioni@unipg.it (L.M.); fiorella.donofrio@studenti.unipg.it (F.D.); katia.fettucciari@unipg.it (K.F.); 2 Department of Pharmacy, University G. d’Annunzio, Via dei Vestini 31, 66100 Chieti, Italy; rosa.amoroso@unich.it

**Keywords:** acetamidine, iNOS inhibitor, nitric oxide, inflammation, pyruvate kinase M2, glycolytic enzymes, mitochondrial function

## Abstract

Neurodegenerative diseases are associated with increased levels of nitric oxide (NO) mainly produced by microglial cells through inducible nitric oxide synthase (iNOS) whose expression is induced by inflammatory stimuli. NO can both exert cytotoxic functions and induce a metabolic switch by inhibiting oxidative phosphorylation and upregulating glycolytic flux. Here, we investigated whether two newly synthesized acetamidine based iNOS inhibitors, namely CM292 and CM544, could inhibit lipopolysaccharide (LPS)-induced BV2 microglial cell activation, focusing on both inflammatory and metabolic profiles. We found that CM292 and CM544, without affecting iNOS protein expression, reduced NO production and reverted LPS-induced inflammatory and cytotoxic response. Furthermore, in the presence of the inflammatory stimulus, both the inhibitors increased the expression of glycolytic enzymes. In particular, CM292 significantly reduced nuclear accumulation of pyruvate kinase M2, increased mitochondrial membrane potential and oxygen consumption rate, and augmented the expression of pyruvate dehydrogenase, pointing to a metabolic switch toward oxidative phosphorylation. These data confirm the role played by NO in the connection between cell bioenergetics profile and inflammation, and suggest the potential usefulness of iNOS inhibitors in redirecting microglia from detrimental to pro-regenerative phenotype.

## 1. Introduction

Nitric oxide (NO) is a radical diffusible compound, which exerts several physiological functions, acting as a biological messenger. NO is biosynthesized by nitric oxide synthase (NOS) which uses l-Arginine as substrate. Three NOS isoenzymes have been identified so far: the constitutive NOS1, or neuronal NOS (nNOS), and NOS3, or endothelial NOS, which are mainly expressed in neurons and vascular endothelium, respectively. The third isoenzyme, known as inducible NOS (iNOS) or NOS2, is inducible and principally expressed by immune cells, including microglial cells [1], where it plays essential roles in inflammation and in the defense against pathogens.

Although NO is deeply connected to the regulation of blood flow and tissue oxygenation, it also acts as neuromodulator and neurotransmitter in the central nervous system (CNS). However, dysregulated NO levels are involved in neurodegenerative and neuroinflammatory diseases [2,3,4]. Microglial cells, the monophagocitic cells of the CNS, respond to an inflammatory stimulus inducing the expression of iNOS that causes an increase in NO levels; this mechanism has been claimed as responsible for the pathogenesis of several neuropathologies, including Alzheimer’s disease, Parkinson’s disease, and amyotrophic lateral sclerosis [5]. Resting ramified microglial cells, activated by immunological stimuli, became amoeboid and upregulate the expression of several proteins, including iNOS, to fulfill a variety of physiological functions, including clearance of toxic cellular debris and immune reactions. However, in pathological conditions, microglial cells can enter a hyperactivation state that induces a detrimental neurotoxic response due to the increase in the production of exceeding NO, reactive oxygen species (ROS), and pro-inflammatory cytokines [3]. It is to highlight that iNOS is deeply involved in the detrimental neurotoxic effects of overactivated microglia. It has been demonstrated that amyloid peptide can induce pro-inflammatory reaction causing neuronal cell death in the presence of microglial cells and that this effect is abolished by iNOS inhibitors [6].

Moreover, NO generated by iNOS is responsible for the inactivation of cytochrome C oxidase, that results in a decrease of adenosine triphosphate (ATP) production via oxidative phosphorylation. This implies that, in order to maintain energy production, NO-producing cells upregulate the glycolytic flux [7,8]. The link between the metabolic pathways used for energy production, i.e., glycolysis or oxidative phosphorylation, and the inflammatory response in immune cells has been strongly suggested. For example, glycolysis promotes proinflammatory activation, whereas oxidative metabolism promotes a less-inflammatory mode of activation in macrophages [9]. Acting as monophagocitic cells in the CNS, also microglial cells may undergo a metabolic switch upon activation. Quiescent microglial cells produce energy mainly via oxidative phosphorylation, whereas upon an inflammatory stimulus, their energy production primarily involves glycolysis [10].

Thus, it is conceivable that the inhibition of NO hyperproduction by iNOS can be a potential therapeutic strategy for neurodegenerative diseases, either because of the removal itself of the pro-oxidant, cytotoxic NO, but also because of the influence on microglial bioenergetic.

**Figure 1:**
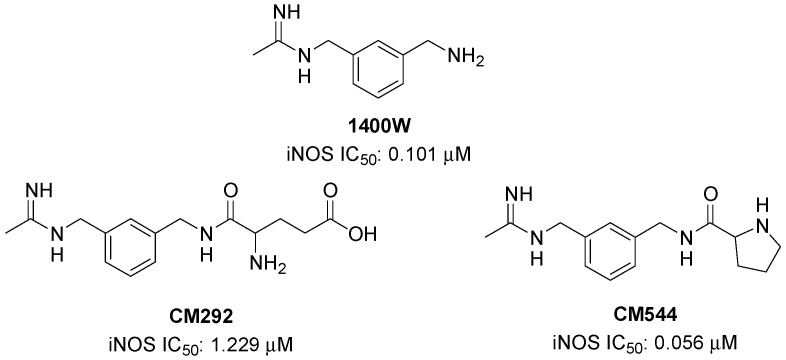
Chemical structure of the evaluated inducible nitric oxide synthase (iNOS) inhibitors.

Several iNOS inhibitors have been disclosed so far for the therapy of different pathological conditions [11,12,13]. In the present study, two iNOS inhibitors, namely CM292 and CM544 (Figure 1), were used to evaluate the potential effects of iNOS inhibition on microglial activation, focusing in particular on both inflammatory and metabolic profiles. Both CM292 and CM544 are acetamidines showing an aminomethylbenzyl core linked to glutamic acid or proline, respectively, which were introduced into the molecular structure in order to exploit the subtle differences among the NOS isoforms at the substrate access channel, and to avoid the unwanted inhibition of the constitutive isoforms [14]. They are structurally related to the commercially available iNOS inhibitor 1400W ((3(aminomethyl)benzyl)acetamidine) (Figure 1) which, however, never passed clinical trials. Very recently, the acetamidine CM544, which is able to inhibit iNOS with a high degree of selectivity with respect to constitutive isoforms (IC_50_ 0.056 μM, eNOS/iNOS selectivity ratio 4583 folds), was studied as potential antiglioma agent [15]. In the present study, the effects of CM292 and CM544 on lipopolysaccharide (LPS) stimulated BV2 cells were investigated in order to assess the potential role of iNOS inhibitors in prompting cell oxidative metabolism for the recovery of a non-inflammatory cell phenotype. We found that, although with different efficacy, both inhibitors counteracted the microglial activation and the metabolic switch induced by lipopolysaccharide (LPS).

## 2. Results

### 2.1. iNOS Inhibitors Reduce NO Production without Affecting iNOS Protein Expression

**Figure 2:**
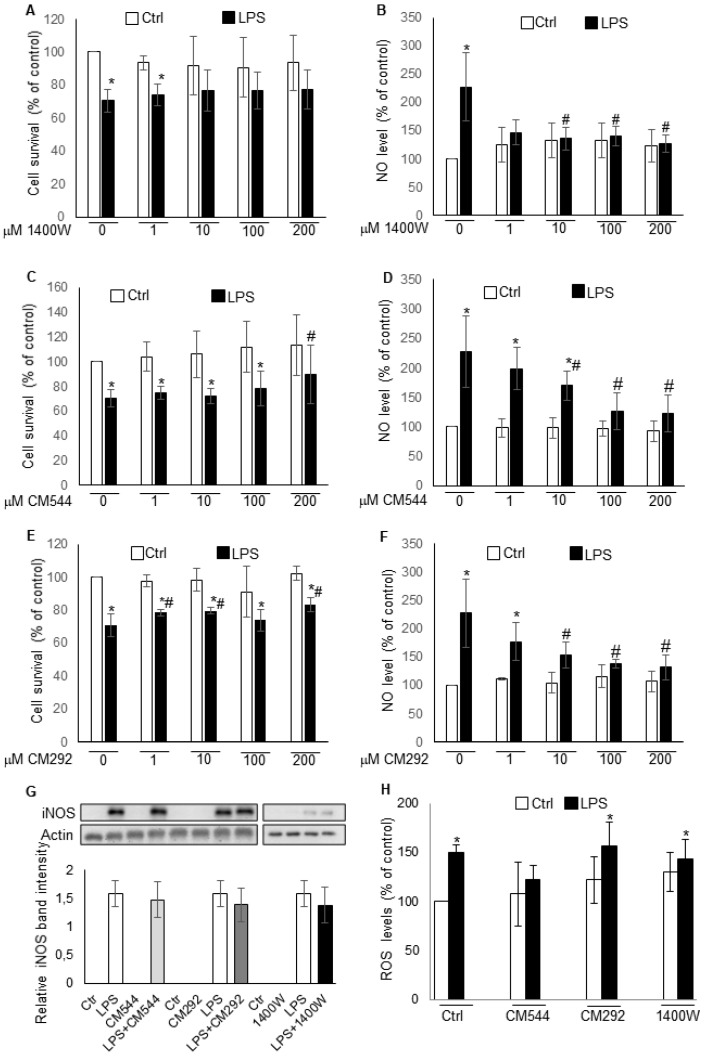
iNOS inhibitors reduce nitric oxide (NO) production without affecting iNOS protein expression. BV2 microglial cells, pre-treated for 3 h with 10 μg/mL LPS, were then exposed for further 24 h to different concentration of iNOS inhibitors and used to determine (**A**,**C**,**E**) cell viability, detected by MTT assay (absorbance of control cells 0.63 ± 0.05 was assumed as 100%). Data represent mean ± SD of *n* = 6 independent experiments performed in quadruplicate. * *p* < 0.05 vs. control cells. # *p* < 0.05 vs. LPS treated cells; (**B**,**D**,**F**) NO production, detected by Griess reagent (absorbance of control cells 1.12 ± 0.25 was assumed as 100%). Data represent mean ± SD of *n* = 6 independent experiments performed in quadruplicate. * *p* < 0.05 vs. control cells. # *p* < 0.05 vs. LPS treated cells; (**G**) Western blotting analysis of iNOS. β-actin was used as loading control. Bars represent the ratio between respective protein and β-actin band intensity. The images are representative of one out of three separate experiments; (**H**) detection of ROS generation by DCF fluorescence (fluorescence of control cells 3.34 ± 0.83 was assumed as 100%). Data represent mean ± SD of *n* = 3 independent experiments performed in quadruplicate. * *p* < 0.05 vs. control cells.

LPS is a typical inflammogen that triggers several microglial responses including an increase in iNOS gene expression leading to the production of NO [16,17,18,19,20]. At low concentrations, NO plays physiological roles in the function of neuronal and vascular cells in CNS, whereas at high concentrations, it is implicated in the pathogenesis of neurodegenerative and neuroinflammatory diseases [2,3,4,5]. To determine whether and how the CM292 and CM544 acetamidine derivatives suppress NO generation, we investigated the effects of these inhibitors on LPS-activated BV2 microglial cells and compared the effects to 1400W. We started this study by determining the effects of 10 μg/mL LPS on cell viability and NO generation. We have previously shown that this LPS concentration is capable of inducing a significant increase in NO generation at the analyzed time point, and that it is suitable for analyzing potential protective and anti-inflammatory effects of therapeutic compounds [21,22]. We found that the 24 h LPS exposure induced a 2.5-fold increase in NO levels and reduced viable cells of approximately 30% (Figure 2A–F). Treatment with 10 μM 1400W reduced LPS-induced NO generation, restoring cell viability (Figure 2A,B). CM292 and CM544 reduced LPS-induced increase in NO levels in a concentration-dependent manner (Figure 2D,F). When used at 100 μM, both inhibitors significantly reduced NO levels. Moreover, when used at 200 μM both inhibitors abolished LPS-induced cytotoxic effects and CM292 restored cell viability to control level (Figure 2F). We also tested CM292 and CM544 on Mouse Embryo Fibroblast (MEFs) and immortalized microglia from hSOD1(G93A) mice (Figure S1). We found that both CM544 and CM292 counteracted LPS induced effects in hSOD1(G93A) microglial cells. Although we could not detect NO production in LPS-treated MEFs, we found that both inhibitors, when used at 100 μM concentration, counteracted LPS-induced cytotoxicity. It is worth noting that both CM292 and CM544 did not affect NO production and cells viability over the considered concentration range (IC_50_ > 200 μM). To verify that the reduction in NO levels was due to the inhibition of iNOS activity and not to an alteration in enzyme expression, we analyzed iNOS protein levels by Western blotting. We found that LPS-induced increase in iNOS protein levels was not affected by the presence of the inhibitors (Figure 2G). These data suggest that the CM292- and CM544- induced NO decrease was due to their ability to inhibit iNOS activity without altering protein expression and are consistent with the effect of the known iNOS inhibitor 1400W. Since LPS increases NO levels by augmenting iNOS expression and ROS levels by activating NOX2 [23], we tested whether CM292 and CM544 could affect ROS production (Figure 2H). We found that a 24 h LPS exposure induced a 1.5-fold increase in ROS levels. As with 1400W, CM292 did not affect LPS-induced ROS production and inhibitors alone did not significantly modify ROS generation, indicating that CM292 did not affect NOX2 activity. It is to note that CM544 lowers LPS-induced ROS generation suggesting that its intracellular action might involve other targets besides iNOS [15].

### 2.2. CM544 and CM292 Modulate the Expression of Glycolytic Enzymes

**Figure 3:**
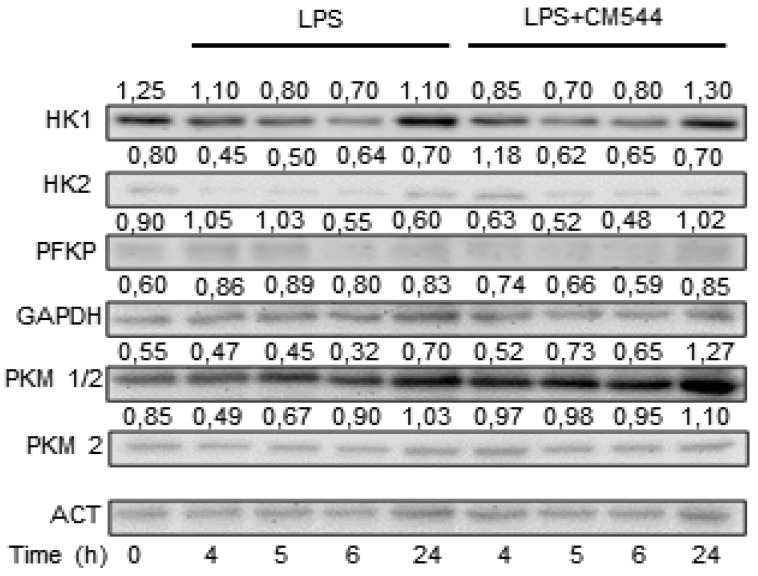
CM544 modulates enzyme of the glycolytic pathway. BV2 microglial cells were pre-treated with 10 μg/mL LPS for 3 h and then 200 μM CM544 was added for further 1 h, 2 h, 3 h, and 21 h. At each time point, cells were collected, and total extract used to analyze the enzyme of the glycolytic pathway by Western blotting. β-actin was used as loading control. Numbers above the images represent the ratio between respective protein and β-actin band intensity. The images are representative of one out of three separate experiments.

It is known that NO determines the inhibition of oxidative metabolism and prevents ATP depletion by glycolysis stimulation [24]. The up-regulation of the glycolytic pathway by LPS was accompanied by increased glucose uptake and expression of glycolytic enzymes in BV2 cells [25]. Thus, we analyzed whether LPS and/or CM544 affect glycolytic pathway in BV2 cells by analyzing the expression levels of several glycolytic enzymes by Western blotting (Figure 3; Figure S2).

We first evaluated hexokinase (HK)1 and HK2, critical enzymes for the maintenance of an elevated glycolytic rate. We found that LPS caused a biphasic response in HK1 protein level, which decreases at early time-points while increasing at 24 h. The presence of 200 μM CM544 did not affect LPS-induced effects on HK1 protein levels. On the other hand, HK2 protein levels were reduced by LPS, whereas short exposure to CM544 increased HK2 expression. We next evaluated phosphofruttokinase (PFK) protein level. PFK is the enzyme that catalyzes the conversion of fructose-6-phosphate to fructose-1,6-bisphosphate, a rate-limiting step in glycolysis. We found that a 6 and 24 h LPS treatment decreases PFK protein level. At an early time-point, the presence of CM544, caused a decrease in PFK protein levels, whereas after a 24 h exposure, PFK expression increased compared to LPS alone. We then analyzed GAPDH protein expression, the first enzyme of pay-off phase of glycolysis. LPS treatment caused a slight increase in GAPDH protein level at each considered time point. CM544 counteracts the effects of the inflammogen restoring GAPDH protein expression to control level after 6 h. Pyruvate kinase is another rate-limiting enzyme in the last step of glycolytic pathway that catalyzes the conversion of phosphoenolpyruvate to pyruvate. Therefore, we investigated whether LPS and CM544 could modulate the expression of two isoforms of this enzyme, i.e., PKM1 and PKM2. The expression of PKM1/2 was slightly modified in the presence of LPS while PKM2 protein level was markedly reduced by the inflammogen after 4 h and 5 h exposures. CM544 upregulated PKM1/2 protein level after 24 h exposure and increased PKM2 protein expression at each considered time-point.

**Figure 4:**
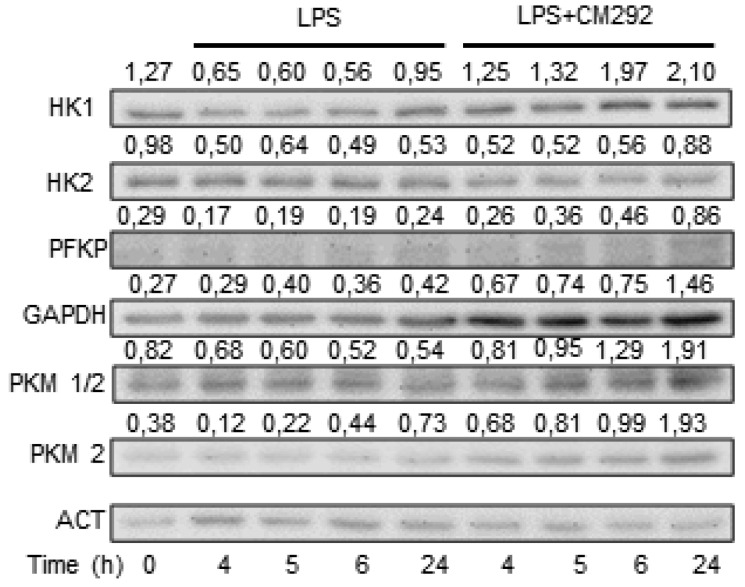
CM292 modulates enzyme of the glycolytic pathway. BV2 microglial cells were pre-treated with 10 μg/mL LPS for 3 h and then 200 μM CM292 was added for further 1 h, 2 h, 3 h, and 21 h. At each time point, cells were collected and total extract used to analyze the enzyme of the glycolytic pathway by Western blotting. Β-actin was used as loading control. Numbers above the images represent the ratio between respective protein and β-actin band intensity. Images are representative of one out of three separate experiments.

We then analyzed whether CM292 could exert any effect on glycolytic pathway enzymes. We found that CM292 counteracted LPS effects on all the analyzed enzyme protein levels, except for HK2 which was only slightly affected after a 24 h exposure (Figure 4; Figure S3). Both CM544 and CM292 alone caused a decrease in HK1 and HK2 protein expression and an increase in PKM2 protein level (Figure S4). These data, indicating that CM544 and CM292 counteract LPS effects, suggest that NO levels can regulate intracellular metabolic flux in microglial cells.

### 2.3. CM292 and CM544 Revert the Effects of LPS on the Metabolic Profile of BV2 Microglial Cells

**Figure 5:**
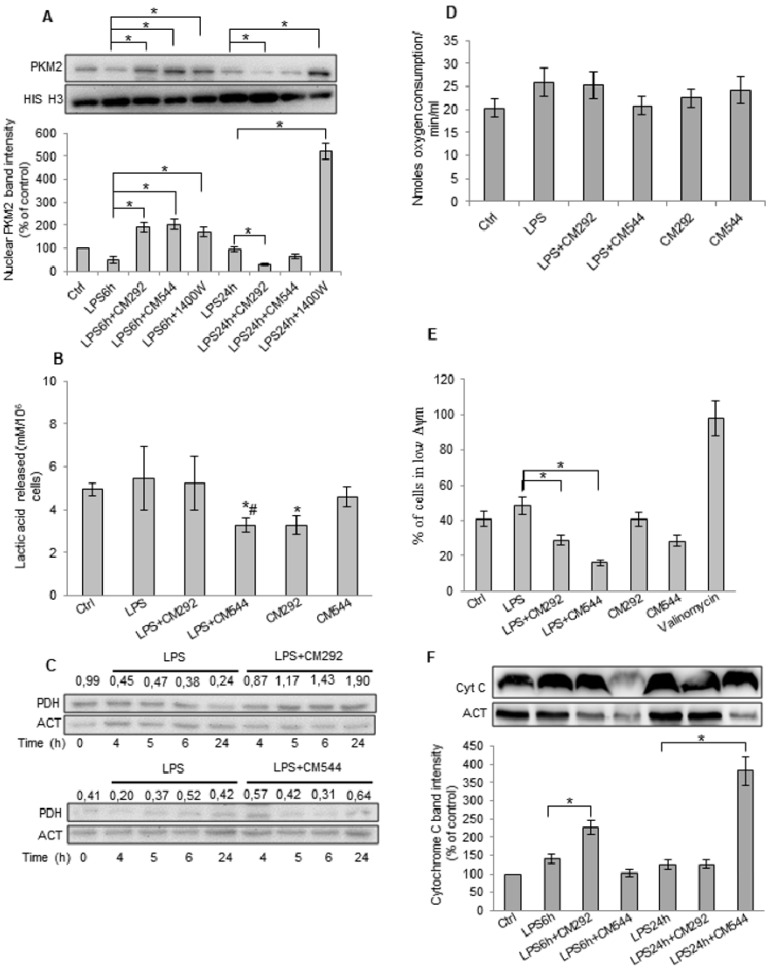
CM292 and CM544 reverted the effects of LPS on the metabolic profile of BV2 microglial cells. BV2 microglial cells were pre-treated with 10 μg/mL LPS for 3 h, and then 200 μM CM292 or CM544 and 10 μM 1400W were added for further for 3 h or 21 h. (**A**) Western blotting analysis of nuclear PKM2 at indicated time. Histone H3 was used as loading control. Bars represent the ratio between respective protein and histone H3 band intensity. The images are representative of one out of three separate experiments; (**B**) lactic acid released after 24 h exposure. Data represent mean ± SD of *n* = 3 independent experiments performed in triplicate. * *p* < 0.05 vs. control cells. # *p* < 0.05 vs. LPS treated cells; (**C**) Time course of pyruvate dehydrogenase (PDH). At each indicated time, cells were collected, and total extract analyzed by Western blotting. β-actin was used as loading control. Numbers above the images represent the ratio between respective protein and β-actin band intensity. The images are representative of one out of three separate experiments. (**D**) Oxygen consumption after 24 exposure. Data represent mean ± SD of n = 3 independent experiments performed in duplicate. * *p* < 0.05 vs. control cells. # *p* < 0.05 vs. LPS treated cells; (**E**) Δψm after 24 h exposure. Bars represent the percentage of cells with low Δψm. (**F**) Western blotting analysis of total cytochrome C. Actin was used as loading control. The images are representative of one out of three separate experiments.

The above data suggest that CM292 and CM544, by decreasing LPS-induced NO generation, modify the bioenergetics profile of BV2 microglial cells. It is reported that NO promotes nuclear translocation of PKM2 [26]. Inside the nucleus, PKM2 works as a protein kinase [27,28] phosphorylating and activating several transcription factors, including HIF-1α and MYC for glycolytic gene expression [29]. Therefore, we investigated whether LPS-induced NO generation in BV2 microglial cells could promote the translocation of PKM2, and whether and how CM292 and/or CM544 could counteract this effect. We found that, after exposure to LPS for 6 h, both inhibitors doubled nuclear translocation of PKM2 compared to the inflammogen alone (Figure 5A). The commercially available inhibitor of iNOS, 1400W, had the same effect of CM292 and CM544. After a 24 h LPS exposure, both CM544 and CM292 determined a reduction in PKM2 nuclear translocation. A 30% and more than 50% decrease was observed in the presence of CM544 and CM292, respectively. It is to note that 1400W dramatically increased PKM2 nuclear translocation, whereas LPS had no effect (Figure 5A). Inhibitors alone caused a decrease in PKM2 nuclear translocation after a 24 h exposure (Figure S5A). These results indicate that both compounds, by decreasing NO-induced PKM2 nuclear translocation, can inhibit glycolytic pathway, reverting the cell to an oxidative metabolism.

To further confirm the effects of iNOS inhibitors on the glycolytic pathway, we measured lactate secretion by BV2 microglial cells after a 24 h LPS exposure (Figure 5B). We found that CM292 did not determine a decrease in lactate secretion compared to LPS-treated cells. Instead, CM544 halved lactate production with respect to the inflammogen, confirming that this inhibitor can decrease the reliance on anaerobic glycolysis. Moreover, with respect to control cells, CM544 alone did not alter lactate secretion, whereas CM292 significantly reduced it. These data indicate that CM544 and CM292 induce a metabolic remodeling and suggest the occurrence of a switch from anaerobic glycolysis to oxidative phosphorylation. Therefore, we evaluated expression of the pyruvate dehydrogenase (PDH), an enzymatic complex that catalyzes the conversion of pyruvate into acetyl-CoA. The results showed that a 24 h LPS exposure decreased PDH protein level of approximately 50%, whereas CM292 increased PDH protein expression (Figure 5C). The exposure to CM544 did not alter PDH expression compared to LPS (Figure 5C). CM544 and CM292 alone did not affect PDH expression (Figure S5B). We then determined mitochondrial oxygen consumption rate (OCR) (Figure 5D) and found that after a 24 h exposure, LPS increased OCR (Figure 5D). CM292 did not modify LPS-induced increase in mitochondrial respiration, whereas CM544 restored it to control values (Figure 5D). Moreover, each inhibitor alone had opposite effects, i.e., CM292 did not modify OCR, whereas CM544 increased it (Figure 5D). To analyze mitochondrial bioenergetics, we next evaluated the effects of the two iNOS inhibitors on mitochondrial membrane potential (Δψm) using a JC-1 probe. LPS treatment for 24 h determined a reduction in mitochondrial membrane potential, as indicated by the increase to about 50% of cell population percentage at low Δψm. CM544 and CM292 counteracted LPS-induced Δψm impairment; the shift of JC-1 towards low Δψm was reduced to 16% and 29%, respectively. In the presence of CM292 and CM544 alone, 30% and 40% of the cells shift towards low Δψm, respectively (Figure 5E). Valinomycin was used as the positive control for Δψm depletion. Moreover, LPS caused a slight increase in cytochrome C expression that, although with a different timing, was further augmented by the inhibitors (Figure 5F). Overall, these results suggest that the iNOS inhibitors, by preventing NO generation, hamper PKM2 nuclear translocation, thus reducing reliance on anaerobic glycolysis and increasing reliance upon oxidative phosphorylation.

### 2.4. CM292 and CM544 Revert LPS-Induced Inflammatory Response

**Figure 6:**
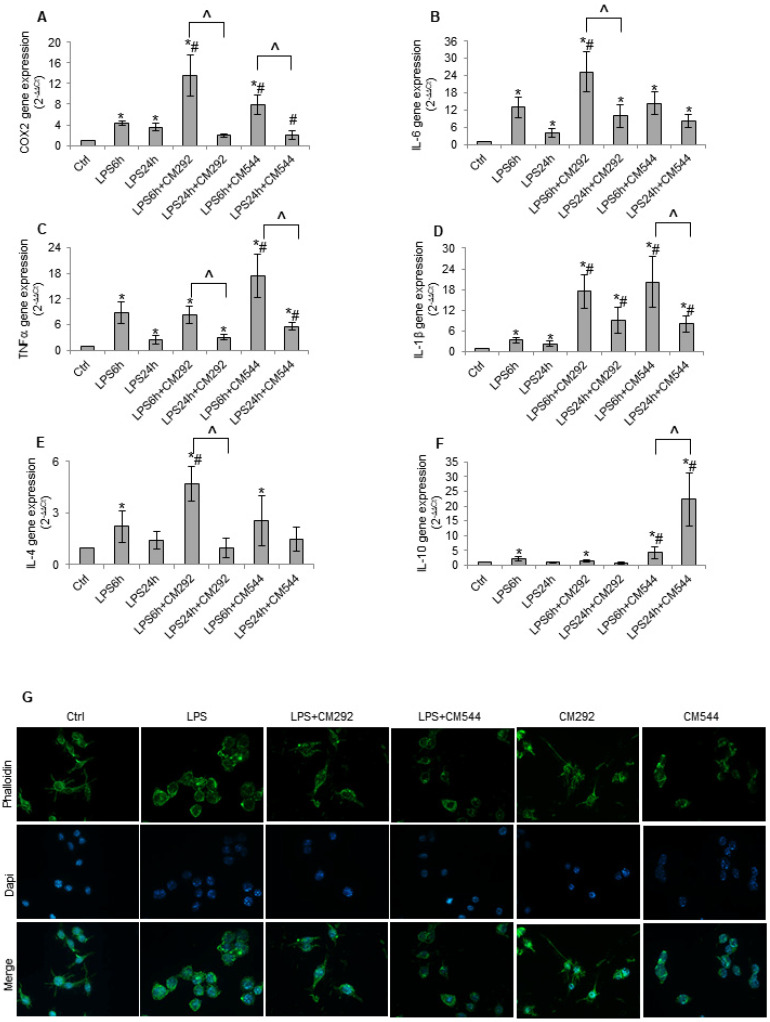
CM292 and CM544 reverted LPS-induced inflammatory response. BV2 microglial cells, pre-treated for 3 h with 10 μg/mL LPS, were then exposed for various time to 200 μM of iNOS inhibitors and used for (**A**–**F**) qRT-PCR of the indicated genes after 6 and 24 h LPS exposure. Gene expression values were normalized to Gapdh and presented as 2^−ΔΔCt^. Relative mRNA gene abundance in untreated cells was assumed to be 1 (control). Data represent mean ± SD of *n* = 3 independent experiments performed in triplicate. * *p* < 0.05 vs. control cells, # *p* < 0.05 vs. LPS treated cells, ^ *p* < 0.05 vs. the respective 6 h treatment; (**G**) Phalloidin staining after a 24 h exposure. The images are representative of one out of three separate experiments. Magnification 40×.

The exposure of BV2 microglial cell to LPS determines a switch from oxidative metabolism towards glycolytic metabolism [25,30]. This bioenergetic remodeling is accompanied by the secretion of pro-inflammatory cytokines like IL-1β, TNFα, and IL-6 [31,32]. The resolution of the inflammatory state is mediated by the release of anti-inflammatory cytokines as IL-4 and IL-10 [33]. Therefore, we evaluated whether CM292 and CM544 could modify gene expression of pro- and anti-inflammatory cytokines. Stimulation with LPS affected the expression of all the considered pro-inflammatory genes. In particular, the expression of IL-6 (Figure 6A), TNF-α (Figure 6B), and IL-1β (Figure 6C) significantly increased after 6 h exposure and was reduced after 24 h of treatment (Figure 6A–C), whereas COX2 gene expression was induced at the same levels both at 6 h and at 24 h (Figure 6D). These results suggest that short exposure to LPS activates acute pro-inflammatory response followed by a shutdown phase of the inflammatory response. CM292 exposure further increased the expression of pro-inflammatory genes (Figure 6A–D); however, after a 24 h exposure, the extent of the reduction was greater than that observed in the presence of the inflammogen alone. Indeed, CM292 determined a reduction in gene expression of IL-1β, TNF-α, and IL-6 of approximately 50% after 24 h. Interestingly, a CM292 exposure for 24 h induced a 76% reduction in COX2 expression restoring control level (Figure 6A–D). Similar results were obtained in the presence of CM544 (Figure 6A–D). The inhibitor halved gene expression of IL-1β e IL-6 and more than halved TNF-α levels after a 24 h LPS exposure. In the presence of CM544, COX2 expression was reduced by 86%, restoring control levels.

The resolution of the inflammatory state is mediated by the switch of microglial cells to an anti-inflammatory phenotype characterized by the release of anti-inflammatory cytokines including IL-4 and IL-10 [33]. LPS induced a significant increase of both cytokines after 6 h treatment that was reduced to control levels at 24 h (Figure 6E,F). After a 6 h LPS exposure, CM292 increased IL-4 gene expression of 40% and did not modify IL-10 gene expression. It is to note that under these experimental conditions, CM292 increased IL-10 gene expression above control levels (Figure 6E,F). On the other hand, in the presence of LPS, CM544 significantly increased IL-10 at both 6 and 24 h, whereas it did not modify IL-4 gene expression at each considered time point (Figure 6E,F). Both inhibitors alone slightly affected all the considered genes (Figure S6). Since both inhibitors reverted NO production to control levels, we wondered whether a difference in the timing of NO generation could be responsible for the observed effects on inflammatory cytokines. To this aim, we treated BV2 cells with LPS for 18 h, a time compatible with NO detection. We then added the inhibitors and followed NO generation over time (Figure S7). In these experimental conditions, we did not find any significant difference in the timing of NO inhibition induced by CM292 and CM544, suggesting that other molecular pathways cooperate with endogenously synthesized NO to counteract inflammatory stimuli. To verify the switch from pro-inflammatory phenotype to anti-inflammatory phenotype, we subsequently determined the effects of both inhibitors on cell morphology. We found that, in the presence of LPS, cells assumed an amoeboid shape characteristic of activated microglia, without evident signs of apoptosis or necrosis. CM292 reverted microglial morphology to the resting shape, confirming that this inhibitor mitigates the pro-inflammatory response (Figure 6G). On the contrary, CM544 was not capable of reverting LPS-induced effects on cell morphology, at least at the analyzed time point (Figure 6G). These results indicate that, although to a different extent, both CM544 and CM292 reduce LPS-induced inflammation.

## 3. Discussion

In this study we show that two newly synthesized compounds, namely CM292 and CM544, reduce LPS-induced NO production in BV2 microglial cells, and, although with different efficacy, they are capable of counteracting LPS-induced microglial activation and metabolic switch induced by LPS.

In physiological conditions, reactive oxygen and nitrogen species (ROS and RNS, respectively) are strictly regulated in order to maintain cell homeostasis. On the contrary, overproduction of reactive species results in damages to cellular components and can alter mitochondrial metabolic processes thus leading to the insurgence of different diseases [34]. The increase in NO levels, primarily mediated by iNOS upregulation, is involved in several neurological disorders such as Alzheimer’s disease, Parkinson’s disease, and amyotrophic lateral sclerosis (ALS) [5,23,31].

The compounds CM292 and CM544 are acetamidine derivatives acting in vitro as specific iNOS inhibitors, and this could represent a potential therapeutic option for the treatment of neurodegenerative diseases. We showed that the two compounds are capable of reverting LPS-induced NO production in immortalized murine BV2 microglial cells. LPS treatment of BV2 cells induces NF-κB activation thus leading to the induction of iNOS expression [21]. We observed that a 3 h LPS exposure was sufficient to increase iNOS protein levels; thus, we chose this time point for inhibitors supplementation. We found that both the compounds reverted LPS-induced increase in NO generation in a dose-dependent manner without affecting iNOS protein levels, thus indicating that the presence of the inhibitors does not alter the enzyme stability. The quite polar profile of the two inhibitors could explain why the significative inhibition of the LPS-induced NO generation was observed only at the evaluated higher doses of 100 μM and 200 μM (see Table S1 in Supplementary Materials). However, it is to highlight that both inhibitors were well tolerated by BV2 cells, since these molecules did not modify cell viability over the evaluated concentration range (IC_50_ > 200 μM), and this observation allowed us to investigate CM292 and CM544 effects at higher doses. In response to immunological stimuli, resting ramified microglial cells are activated to an amoeboid morphology and upregulate several molecules that span from iNOS to cyclo-oxygenase-2 (COX-2) and pro-inflammatory cytokines in order to fulfill physiologic tasks within the CNS [3]. We showed that BV2 cells acquired an amoeboid shape in the presence of LPS that was completely reversed to a ramified morphology by CM292. The protective effects of CM292 also culminate in the abolishment of LPS-induced cell death, thus suggesting a potential cytoprotective function for the inhibitor, at least toward a pro-inflammatory insult. On the other hand, the incubation with CM544 did not revert amoeboid phenotype, at least at the analyzed time point. It is worth noting that both CM292 and CM544 induced an increase in the expression of pro-inflammatory cytokines at early time points. This effect was then followed by a sharp decrease after a 24 h exposure. In particular, in the presence of CM292 and CM544, IL-1β levels were more than halved and COX2 gene expression returned to control levels. These data suggest that both the inhibitors exert an initial pro-inflammatory action, followed by a rapid inhibition of the inflammatory response, that culminates, at least for CM292, in the re-acquisition of a ramified/resting phenotype. These results recapitulate the concept of hormesis, i.e., an adaptive response of cells to a moderate stress [35,36]. A similar behavior has been reported for epigallocatechin 3-gallate (EGCG), an antioxidant extensively studied for its beneficial health effects. However, an outcome of EGCG treatment is the production of hydrogen peroxide and hydroxyl radicals. Thus, EGCG can exert its antioxidant function via an initial pro-oxidant behavior at least in some cellular contexts [37].

CM292 and CM544 are both capable of restoring NO to control levels, thus we analyzed whether the reduced amount of available NO could exert metabolic functions. It has been thoroughly demonstrated that NO can induce a metabolic switch in immune cells by inhibiting cytochrome C oxidase [7,38]. This effect culminates in the inhibition of mitochondrial oxidative phosphorylation. Thus, in the presence of an excess of NO, cells should rely on anaerobic glycolysis to obtain the necessary amount of energy to survive and accomplish physiological functions [10]. We first examined the expression of key glycolytic enzymes and found that, in the presence of the inhibitors, almost all the analyzed proteins were upregulated, indicating an increase in the glucose flux through the glycolytic pathway.

In order to understand whether CM292- and CM544-induced decrease in NO levels could result in a metabolic switch, we focused our attention on the glycolytic enzyme PKM2, since it is a critical modulator of glycolytic reprogramming in LPS-stimulated macrophages [32]. In its dimeric form PKM2 can translocate to the nucleus, where it induces the glycolytic reprogramming by stabilizing Hif-1α [32]. On the other hand, cytoplasm-residing tetrameric PKM2 reverses LPS-induced pro-inflammatory traits [32]. It has also been reported that iNOS-derived NO could promote a glycolytic switch by inducing nuclear translocation of PKM2 [26]. We found that, after an initial increase in PKM2 nuclear translocation, both the inhibitors strongly reduced nuclear accumulation of PKM2, and for CM292 this effect was also statistically significant. In order to decipher the implication of PKM2 nuclear translocation on BV2 cells metabolic behavior, we analyzed mitochondrial function in terms of oxygen consumption rate (OCR) and mitochondrial membrane potential (Δψm) and measured lactic acid release in the extracellular milieu. When analyzing the effects of CM544 in pro-inflammatory conditions, we found that, although it increased mitochondrial membrane potential, it induced a decrease in OCR. These results are in accordance with the observation that PKM2 nuclear translocation is not reduced in a statistically significant manner. Moreover, CM544 did not modify the expression of PDH which is responsible for the conversion of glycolysis-derived pyruvic acid into acetyl-CoA for its entrance in the tricarboxylic acids cycle. We also observed that, in a pro-inflammatory condition, CM544 induced an upregulation of HK2, while sparing HK1. This is in accordance with the nuclear levels of PKM2 observed in the same experimental conditions. In fact, nuclear PKM2 can behave as a protein kinase acting on STAT3 [29]. Activated STAT3, in turn, selectively induces the expression of HK2. It has been demonstrated that reliance on glycolysis is essential for microglial activation, and that HK2 specifically drives inflammatory responses [39]. Taken together, these data point to a low efficacy of CM544 on the alteration of metabolic behavior due to the prolonged presence of PKM2 in the nuclear compartment, coherently with the absence of morphology reversion.

On the contrary, we found that in a pro-inflammatory condition CM292 increased Δψm and OCR without affecting lactic acid release in the extracellular milieu. These data, in line with the decrease in nuclear PKM2 accumulation, are further strengthened by the increase in the expression of PDH. Altogether, the obtained results point to a metabolic switch toward oxidative phosphorylation in the presence of CM292, which is strictly correlated to the observed restoration in cell morphology.

Although both CM292 and CM544 reduce NO levels of the same magnitude and with overlapping timing, different reasons could be at the basis of their slightly divergent mode of action. It is plausible that the compounds could bind to further targets besides iNOS; indeed, in a previous study, CM544 stimulated Nrf-2 expression in C6 rat glioma cells [15]. However, the assayed iNOS inhibitors could trigger a different toxicological cellular response, which, although without impairing cells survival, can alter the same metabolic switch. Indeed, it can be observed that in LPS treated cells, TNFα gene expression is higher in the presence of CM544 compared to CM292, and also IL10 gene expression is quite enhanced after 24 h. A further explanation of the different effects of the two molecules could lay in their supposedly different pharmacokinetic profiles, although no in vivo data are currently available. In fact, CM292 and CM544 are quite polar compounds, with similar predicted lipophilicity and BCF, although slightly higher for CM292 (see Table S1 in Supplementary Materials). Thus, a better biodistribution could be supposed for CM292 with respect to CM544, although more investigations are needed to assess the pharmacokinetic, and to shed light on the reasons for the observed differences on the metabolic switch.

## 4. Conclusions

In the present study, two newly synthesized iNOS inhibitors, i.e., CM292 and CM544, were used to investigate the potential usefulness of iNOS inhibition in the recovery of oxidative metabolism in LPS stimulated BV2 cells. Although both the compounds inhibit iNOS enzymatic activity leading to a significant reduction in NO levels, CM292 seems to exert a stronger metabolic action that relies on a decrease in PKM2 nuclear translocation. Moreover, since activated microglia augments reliance on glycolysis by increasing lactate production and decreasing mitochondrial oxygen consumption, it appears that CM292, possibly by reverting these metabolic actions, can also revert microglial activation, as confirmed by the reacquisition of the ameboid morphology. It is to underline that CM292 also exerts cytoprotective effects toward LPS-induced cytotoxicity. In general, these results confirm the iNOS involvement in driving microglia bioenergetics, and suggest that iNOS inhibition could be a strategy to restore resting microglia phenotype. At the same time, the collected results prompt the research of new potent and selective iNOS inhibitors able to affect cell energetic metabolism, and potentially useful to treat neuroinflammatory diseases.

## 5. Material and Methods

### 5.1. Materials

All the reagents, unless otherwise stated, are from Merck KGaA (Darmstadt, Germany). iNOS inhibitors CM292 and CM544 were prepared according to the given procedure [14]. Flash chromatography was performed on silica gel 60 and TLC on silica gel 60, F254.

### 5.2. General Procedure for the Synthesis of CM292 and CM544

The synthesis of compounds CM292 and CM544 started dissolving, respectively, Boc-l-glutamic acid 5-tert-butyl ester or *N*-(tert-Butoxycarbonyl)-l-proline (0.002 mol) in dry DMF (4 mL); then, NMM (0.002 mol) was added under N_2_. After 5 min under stirring at −10 °C, isobutylchloroformate was added (0.002 mol) under N_2_ and the mixture reacted for 10 min. Then a solution of 1400W [40] (0.0025 mol) in dry DMF (3 mL) was added under N_2_, and after 20 h at 0 °C and 4 h at room temperature, reaction was quenched with water. The solvent was removed under reduced pressure, and the crude product was purified by column chromatography on silica gel (dichloromethane/methanol, 9:1). The purified compound (0.002 mol) was then dissolved in a mixture of dioxane (15 mL) and HCl 4N (2 mL) and stirred for 24 h. Then the solvent was evaporated under vacuum and the solid residue was dissolved in H_2_O and washed with ethyl acetate (3 × 10 mL). The aqueous layer was freeze-dried obtaining the desired compound CM292 or CM544 (for chemical characterization see supporting information).

### 5.3. Cell Cultures and Treatments

BV2 microglial cells were cultured in RPMI supplemented with 5% foetal bovine serum (FBS), glutamine (4 mM), penicillin (50 U/mL), and streptomycin (50 mg/mL) at 37 °C in a humidified 5% CO_2_ environment and seeded at the density of 250,000/cm^2^. After 24 h subculture, cells were treated with 10 μg/mL LPS [21,22] for 3 h and then exposed to iNOS inhibitors in the presence of LPS. The inhibitors were added for 1 h, 2 h, 3 h, and 21 h resulting in 4 h, 5 h, 6 h, and 24 h LPS plus inhibitor treatment time.

### 5.4. Cell Viability

Live cells were counted by hemocytometer and viability assessed by the conventional MTT (3-[4,5-dimethylthiazol-2-yl]- 2,5-dephenyl tetrazolium bromide) reduction assay after washing the cells with PBS. To ensure that results were comparable between cell counting methods, calibration curves for MTT absorbance vs. cell numbers in parallel cultures were constructed. The relationship between absorbance and cell number was found to be linear. Results were expressed as the percentages of reduced MTT, assuming the absorbance of control cells as 100%.

### 5.5. Measurement of NO Production

Nitric oxide (NO) production was determined indirectly through the measurement of nitrite, a stable metabolite of nitric oxide, by Griess reaction. After the treatments, a 50 μL aliquot of culture medium was mixed with an equal volume of Griess reagent, incubated for 20 min at room temperature and absorbance read at 550 nm using a microplate reader (Tecan, Hombrechtikon, Switzerland). Results were expressed as percentage of the control, assumed as 100%. Nitrite standard reference curve was prepared for each determination.

### 5.6. ROS Production

The 2′,7′-dichlorodihydrofluorescein diacetate (DCFH-DA) method was used to detect ROS intracellular levels. Cells were loaded with DCFH-DA (10 μM), which was added into the medium for a further 30 min at 37 °C. The fluorescence of 2′,7′-dichlorofluorescein was detected at 485 nm excitation and at 535 nm emission, using a TitertekFluoroscan II (Flow Laboratories, McLean, VA. USA). Results were expressed as the percentage of the control DCF fluorescence.

### 5.7. Real Time PCR

Total RNA was isolated with TRIZOL Reagent (Invitrogen Ltd., Paisley, UK) according to the manufacturer’s instructions, and cDNA was synthesized using the iScript cDNA synthesis kit (Bio-Rad Lab, Hercules, CA., USA). Real-time PCR was performed using the iCycleriQ detection system (Bio-Rad) and SYBR Green chemistry. Mouse primer sequences were listed in Table 1. SYBR Green RT-PCR amplifications were carried out in a 96-well plate in a 25 μL reaction volume that contained 12.5 μL of 2×iQ™ SYBR^®^ Green SuperMix (Bio-Rad), 400 nM forward and reverse primers, and 5–40 ng of cDNA. In each assay, no-template controls were included, and each sample was run in triplicates. The thermal profile consisted of incubation at 95 °C 3 min, followed by 40 cycles of denaturation for 10 s at 95 °C and an annealing/extension step of 30 s at 62 °C. Mean of Ct values of the stimulated sample was compared to the untreated control sample. ΔCt is the difference in Ct values derived from the target gene (in each assayed sample) and Gapdh, while ΔΔCt represents the difference between the paired samples. The n-fold differential ratio was expressed as 2^−ΔΔCt^.

### 5.8. Western Blotting Analyses

Cells were lysed in boiling Laemmli sample buffer or processed with NE-PER(R) Nuclear and Cytoplasmic Extraction Reagents (Pierce Biotechnology, Rockford, IL, USA) according to manufacturer’s instruction. Extracts were loaded on SDS-polyacrylamide gel, transferred on nitrocellulose membrane, and immunoblotted with antibodies listed in Table 2. β-actin antibody and Histone H3 antibody were used as marker proteins for total and nuclear extracts, respectively. Immunocomplexes were visualized with an enhanced chemiluminescence kit (ECL, Pierce Biotechnology, Rockford, IL) and acquired with ChemiDoc Imaging System (Bio-Rad) applying a fixed exposure time for each antibody. Band intensity was analyzed by ImageJ software.

### 5.9. Actin Labeling

Phalloidin was used to detect filamentous actin (F-actin) content in BV2 microglial cell. The cells were fixed with 4% PFA for 20 min at room temperature and F-actin was stained with fluorescein isothiocyanate (FITC) labelled phalloidin (1:250) for 30 min at room temperature. Cell nuclei were counterstained with 4′, 6′-diamidino-2phenylindole (DAPI), and samples were analyzed with a Zeiss Axio Observer Z1 equipped with Apotome and digital Camera AxiocamMRm (Zeiss, Oberkochen, Germany).

### 5.10. Lactate Determination

Lactate quantification was obtained by using enzymatic spectrophotometric method. BV2 cell culture medium was treated with TCA (10% *v*/*v*) and centrifuged at 1200× *g* for 5 min. Supernatant was incubated with LDH reaction buffer (280 mM hydrazine, 467 mM glycine, 2.6 mM EDTA, 2.5 mM β-NAD^+^, 500 units L-lactic dehydrogenase) and spectrophotometrically monitored.

In this enzymatic method, lactate is oxidized into pyruvic acid by lactate dehydrogenase (LDH) using NAD^+^ as cosubstrate/coenzyme, which is reduced to NADH+H^+^. The reduced NADH absorbs at 340 nm, which is directly proportional to lactate concentration. Results were expressed as mM of lactate released/10^6^ cells. Lactate standard curve was prepared for each determination.

### 5.11. Analysis of Mitochondrial Membrane Potential

Mitochondrial membrane potential (Δψm) was determined by using the JC-1 fluorescent probe (6 μM) added to cells 30 min before the end of the incubation. For complete depletion of Δψm (positive control), the mitochondrial ionophore valinomycin (1 μM) was used. Cells were recovered and changes of Δψm were analysed using an EPICS XL-MCL (Beckman Coulter) using 488 nm excitation with 525 nm and 575 nm bandpass emission filters.

### 5.12. Oxygen Consumption Rate

The rate of oxygen consumption in intact cells was determined as previously described [41]. Briefly, after treatment, 6 × 10^6^ BV2 cells were resuspended in 1 mL of respiration buffer (137 mM NaCl, 5 mM KCl, 0.7 mM NaH_2_PO_4_, 10 mM glucose, and 25 mM Tris-HCl, pH 7.4). Oxygen concentration was recorded into a thermostatic chamber at 37 °C with stirring. The medium contained about 200 nmol oxygen/mL at 37 °C.

### 5.13. Statistical Analysis

Data are expressed as mean ± SD. Differences between groups were identified using one-way ANOVA followed by Dunnet’s post-hoc test (with control or LPS as reference condition). Values with *p* values less than 0.05 were considered significant. Data analyses were performed using Prism software.

## Figures and Tables

**Table 1:** List of primers.

Gene Name	Gene Symbol	Primer Sequences (F: Forward R: Reverse)
Glyceraldehyde-3-phosphate dehydrogenase	GAPDH	F:GCCAAATTCAACGGCACAGT R:AGATGGTGATGGGCTTCCC
Interleukin 1beta	IL-1β	F:AAAAGCCTCGTGCTGTCGGACC R:TTGAGGCCCAAGGCCACAGGT
Interleukin 6	IL-6	F:GCTGGAGTCACAGAAGGAGTGGC R:GGCATAACGCACTAGGTTTGCCG
Interleukin 4	IL-4	F:TCCGATTCCTGAAACGGCTC R:CAACGTACTCTGGCTGGCT
Interleukin 10	IL-10	F:CAGCAGTGCTATGCTGCCTGCT R:GTGGCTCTGGCCGACTGGGA
Tumor necrosis factor alpha	TNF-α	F:GCCCACGTCGTAGCAAACCAC R:GGCTGGCACCACTAGTTGGTTGT
Cyclooxygenase 2	Cox-2	F:AAGACTTGCCAGGCTGAACT R:CTTCTGCAGTCCAGGTTCAA

**Table 2:** List of Antibodies.

9	Abbreviation	Molecular Weight (kDa)	Dilution	Supplier
Actin	ACT	43	1:400	Santa Cruz Biotechnology Santa Cruz, CA
Cytochrome C	CytC	12.5	1:500	Santa Cruz BiotechnologySanta Cruz, CA
Glyceraldehyde 3-phosphate dehydrogenase	GAPDH	37	1:1000	Cell SignalingTecnology, Danvers, MA
Hexokinase I	HK1	102	1:1000	Cell SignalingTecnology, Danvers, MA
Hexokinase II	HK2	102	1:1000	Cell SignalingTecnology, Danvers, MA
Histone H3	His H3	17	1:200	Santa Cruz BiotechnologSanta Cruz, CA
Inducible nitric oxide synthase	iNOS	103	1:400	Sigma AldricSt. Luis, MO
Phosphofructokinase Platelet	PFKP	80	1:1000	Cell SignalingTecnology, Danvers, MA
Pyruvate Dehydrogenase	PDH	43	1:1000	Cell SignalingTecnology, Danvers, MA
Pyruvate Kinase M1/M2	PKM1/2	60	1:1000	Cell SignalingTecnology, Danvers, MA
Pyruvate Kinase M2	PKM2	60	1:1000	Cell SignalingTecnology, Danvers, MA

## Supplementary Materials

The following are available online, General methods and materials. Mouse Embryo Fibroblast (MEFs) culture and treatment. Immortalized microglia culture and treatment. Chemical characterization of CM292 and CM544. Figure S1: Supplemental to Figure 2. Figure S2: Supplemental to Figure 3. Figure S3: Supplemental to Figure 4. Figure S4: Supplemental to Figure 3 and Figure 4. Figure S5: Supplemental to Figure 5. Figure S6: Supplemental to Figure 6. Figure S7: Supplemental to Figure 6. Table S1: Calculated lipophilicity and bioconcentration factor of CM292 and CM544.
