# Supplementary material for: Acetamidine-Based iNOS Inhibitors as Molecular Tools to Counteract Inflammation in BV2 Microglial Cells
Source: Molecules. 2020 Jun 6;25(11):2646. doi: 10.3390/molecules25112646 (PMC7321217; doi:10.3390/molecules25112646)
Supplement: Supplementary file 1 [file molecules-25-02646-s001.pdf]

Supplementary material

Acetamidine based iNOS inhibitors as molecular tools to counteract inflammation in BV2 microglial cells

Silvia Grottelli[1], Rosa Amoroso[2], Lara Macchioni[1], Fiorella D'Onofrio[1], Katia Fettucciari[1], Ilaria Bellezza[1,a,*] and Cristina Maccallini[2,a,*].

[1] University of Perugia, Department of Experimental Medicine, Polo Unico Sant'Andrea delle Fratte, 06132, Perugia, Italy.
[2] University G. d'Annunzio, Department of Pharmacy, Via dei Vestini 31, 66100, Chieti, Italy.

[a] These Authors share the senior authorship

***** Correspondence: Cristina Maccallini, PhD: cristina.maccallini@unich.it; Ph.: +39 08713554693; Ilaria Bellezza, PhD: ilaria.bellezza@unipg.it; Ph: +39 075 585 8226

Contents

General methods and materials

Mouse Embryo Fibroblast (MEFs) culture and treatment

Immortalized microglia culture and treatment

Chemical characterization of CM292 and CM544

Figure S1, S2, S3, S4, S5, S6; S7

Table S1

General methods and materials

All chemicals were purchased from commercial sources and used without further purification. Infrared spectra were recorded on a FT-IR 1600 Perkin-Elmer spectrometer. NMR spectra were run on a Varian instrument, operating at 300 (^{1}H) or 75 (^{13}C) MHz; chemical shifts (δ) are reported in ppm. HPLC analyses were performed using a Waters (Milford, MA, USA) system composed of a P600 model pump, a 2996 photodiode array detector, and a 7725i model sample injector (Rheodyne, Cotati, CA, USA). Chromatograms were recorded on a Fujitsu Siemens Esprimo computer and the Empower Pro software (Waters) processed data. The analyses were performed on an XTerra MS C8 column (250 x 4.6 mm id, 5 μm particle size) (Waters), equipped with an XTerra MS C8 guard column (Waters). A column thermostat oven module Igloo-Cil (CilCluzeau Info Labo, France) was used. To evaluate target compounds purity, the column was eluted at a flow rate of 1 mL/min with a mixture of 8 mM sodium borate (pH = 10) and CH_3OH (mobile phase composition ranging from 50/50 to 75/25). All tested compounds had a purity of ≥95%. Elemental analyses were carried out by a Eurovector Euro EA 3000 model analyzer. Analyses indicated by the symbols of the elements were within ± 0.4% of the theoretical values.

Mouse Embryo Fibroblast (MEFs) culture and treatment

MEFs were prepared from individual embryos at embryonic day13.5 (E13.5). MEFs were maintained in DMEM containing 10% FBS glutamine (4 mM), penicillin (50 U/ml), and streptomycin (50 mg/ml) at 37 ℃ in a humidified 5% CO2 environment and seeded at the density of 300,000/cm2. After 24 h subculture, cells were treated with 5μg/ml LPS for three hours and then exposed to iNOS inhibitors in the presence of LPS for 24h.

Immortalized microglia culture and treatment

Immortalized microglial cells, obtained from embryonic (E14) cortices from hSOD1G93A mice according to Righi and colleagues, were a kind gift of Dr. G. Pietrini (Università di Milano). Microglia were then characterized by Western blot and immunofluorescence for the presence of selective markers (colony stimulating factor 1, CSF-1) and the absence of astrocyte-specific molecules (i.e., glial fibrillary acidic protein, GFAP). hSOD1G93A microglial cells were cultured in DMEM F12 supplemented with 5% Foetal Bovine Serum (FBS), glutamine (4 mM), penicillin (50 U/ml), and streptomycin (50 mg/ml) at 37℃ in a humidified 5% CO2 environment. After 24 h subculture, cells were treated with 1μg/ml LPS for three hours and then exposed to iNOS inhibitors in the presence of LPS for 24h.

Chemical characterization of CM292 and CM544

{1-[(3-{[(2-ammonio-4-carboxybutanoyl)amino]methyl}benzyl)amino]ethylidene}ammonium dichloride (**CM292**)

Yellow sticky solid, 95% yield. IR (KBr): 3396, 3089, 2915, 1681; ^{1}HNMR (300 MHz, DMSO) δ 1.90 (m, 2H, CH_2), 2.12 (s, 3H, CH_3), 2.24 (t, J=6.9 Hz, 2H, CH_2), 3.77-3.82 (m, 1H, CH), 4.14-4.39 (m, 4H, CH_2), 7.10-7.28 (m, 4H, CHAr), 8.35 (s, 3H, NH), 8.82 (s, 1H, NH), 9.17 (t, J= 5.7, 1H, NH), 9.26 (bs, 1H, NH), 9.99 (bs, 1H, NH). ^{13}CNMR (75 MHz,DMSO) δ 19.2, 26.9, 29.8, 42.6, 45.5, 52.2, 127.1, 127.2, 127.4, 129.2, 136.2, 139.6, 164.6, 168.8, 173.8. Anal. calcd. for $C_{15}H_{24}Cl_2N_4O_3$: C 47.50, H 6.38, N 14.77; found C 47.59, H 6.36 N 14.62.

2-{[(3-{[(1-iminioethyl)amino]methyl}benzyl)amino]carbonyl}pyrrolidinium dichloride (**CM544**)

Pale yellow sticky solid. Yield 70%. IR (KBr):3379, 3108, 2914, 1681 cm^{-1}. ^{1}HNMR (300 MHz, DMSO) δ: 1.84 (m, 2H, CH_2), 2.20 (s, 3H, CH_3), 3.19 (q, J= 5.1 Hz, 1H, CH), 3.24 (pentet, J= 6.3 Hz, 4H, CH_2), 4.33 (d, J= 5.7 Hz, 2H, CH_2), 4.48 (d, J= 6.0 Hz, 2H, CH_2), 7.20-7.36 (m, 4H, CHAr), 8.81 (bs, 3H, NH), 8.93 (s, 1H, NH), 9.31 (t, J=6.3 Hz, 2H, NH), 10.09 (bs, 1H, NH); ^{13}CNMR (75 MHz,DMSO) δ: 19.2, 24.3, 30.2, 42.8, 46.6, 59.5, 63.4, 127.1, 127.2, 129.9, 136.3, 139.7,164.8, 168.7 Anal. calcd. for $C_{15}H_{24}Cl_2N_4O$: C 57.78, H 7.76, N 17.97; found: C 57.82, H 7.71, N 17.90.

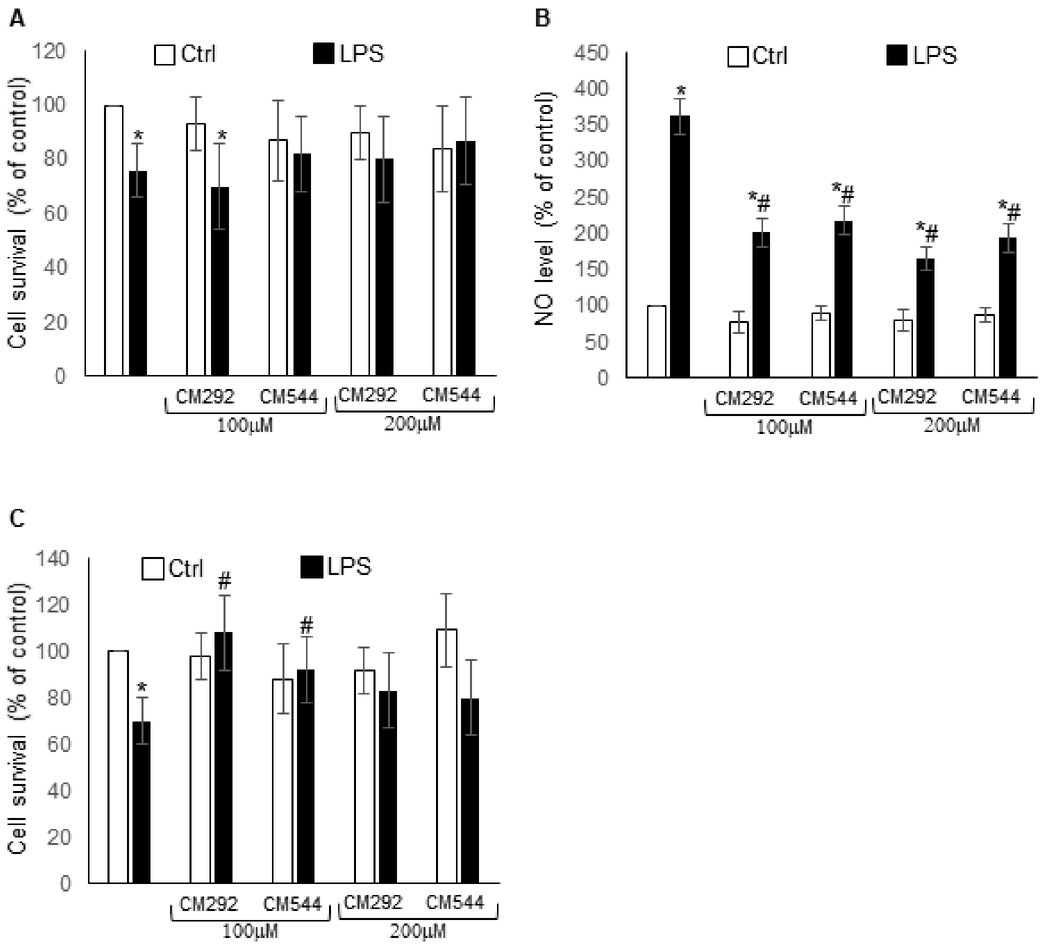

Figure S1. Supplemental to Fig. 2.

hSOD1(G93A) microglial cells (A and B) and MEFs (C), pre-treated for 3h with 1µg/ml and 5µg/ml LPS, respectively, were then exposed for another 24h to different concentrations of iNOS inhibitors and used to determine cell viability, detected by MTT assay and NO production, detected by Griess reagent. Data represent mean ± SD of n=3 independent experiments performed in quadruplicate. *$p < 0.05$ vs control cells. #$p < 0.05$ vs LPS treated cells.

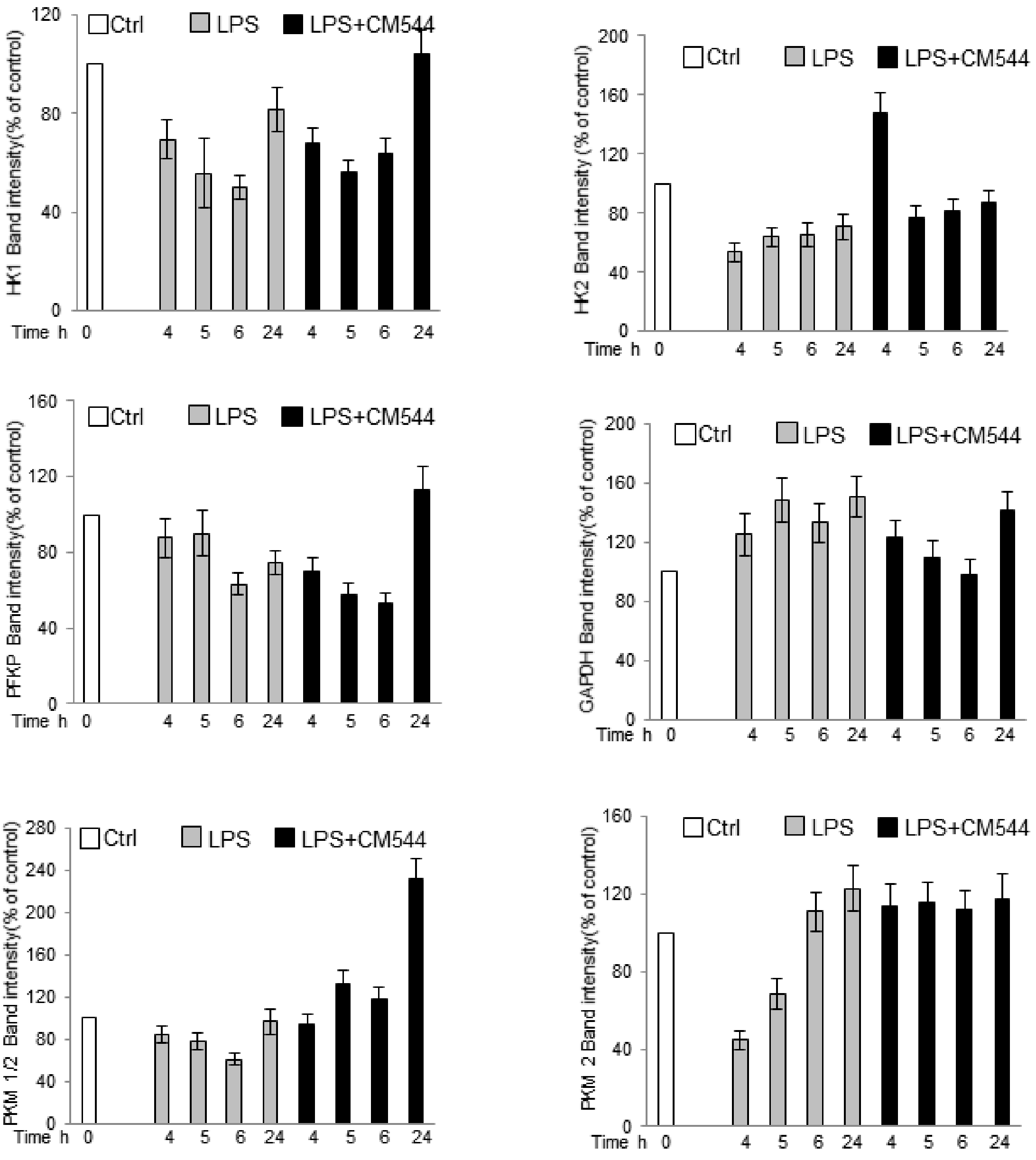

Figure S2. Supplemental to Fig. 3. Graphical representation of densitometric analyses of the proteins detected by western blotting and reported in Fig. 3. Each band was normalized to β–actin and the densitometric value of untreated cells was assumed as 100%.

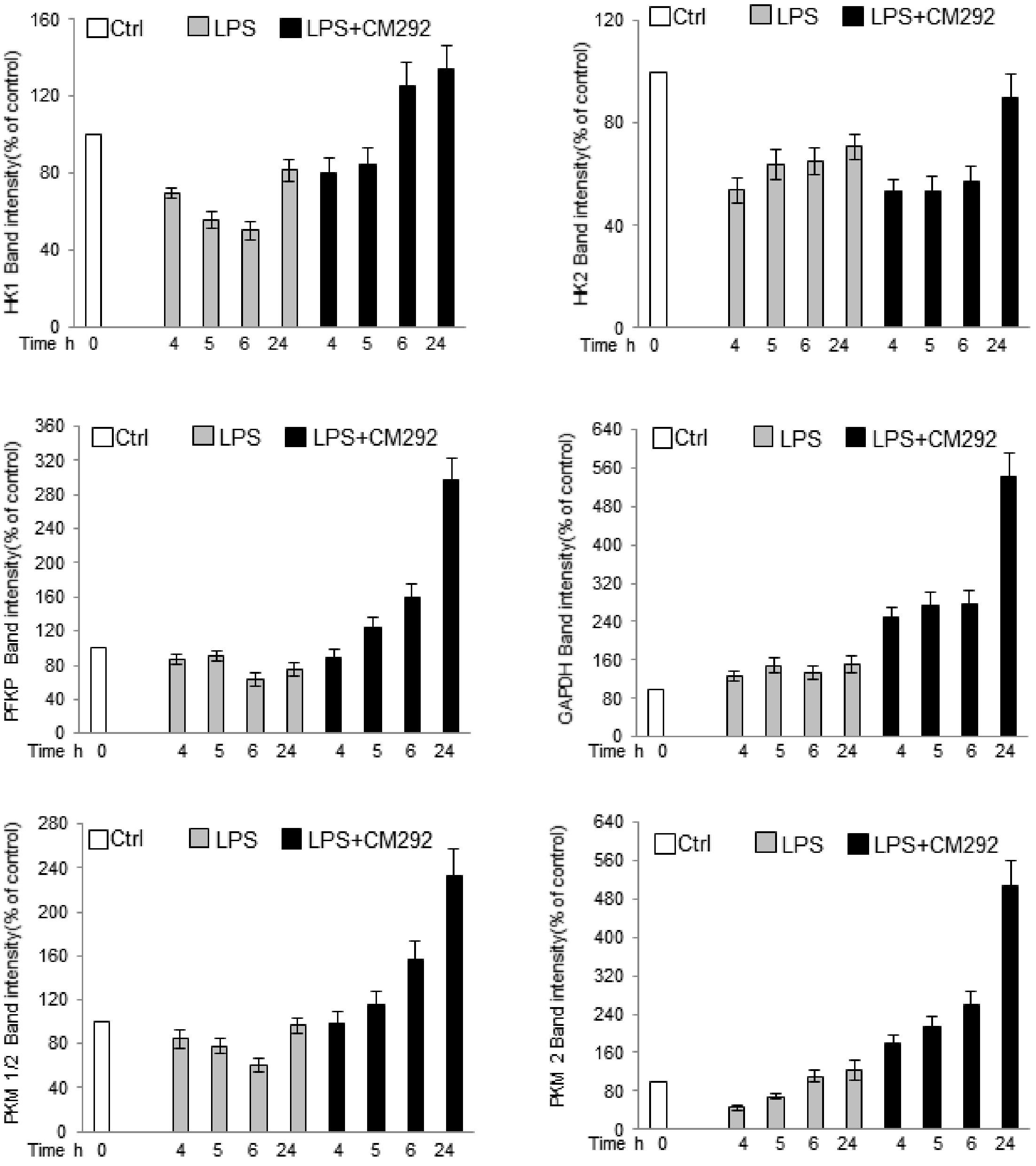

Figure S3. Supplemental to Figs. 4 Graphical representation of densitometric analyses of the proteins detected by western blotting and reported in Fig. 4. Each band was normalized to β–actin and the densitometric value of untreated cells was assumed as 100%.

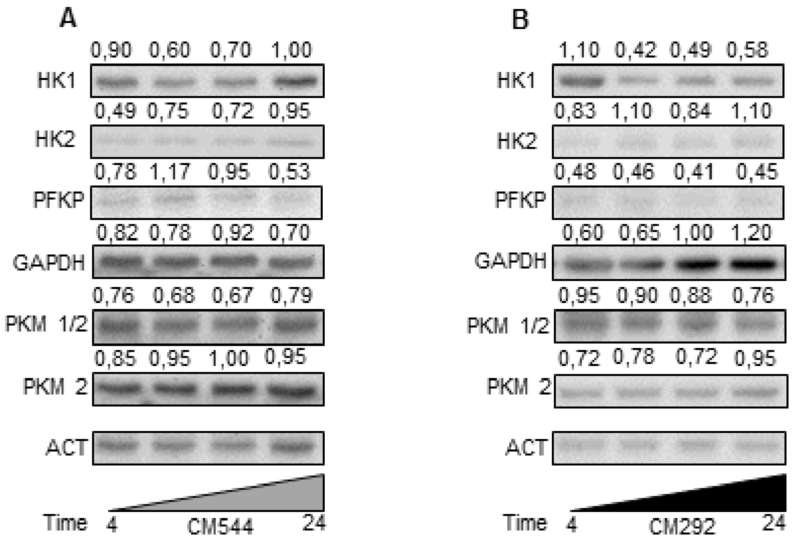

Figure S4. Supplemental to Figs. 3 and 4. BV2 microglial cells were treated with 200µM CM544(A) or 200µM CM292, (B) for various time. At the end of treatment, cells were collected, and total extract used to analyze the enzyme of the glycolytic pathwayby Western Blotting. β–actin was used as loadingcontrol. The images are representative of one out of three separateexperiments.

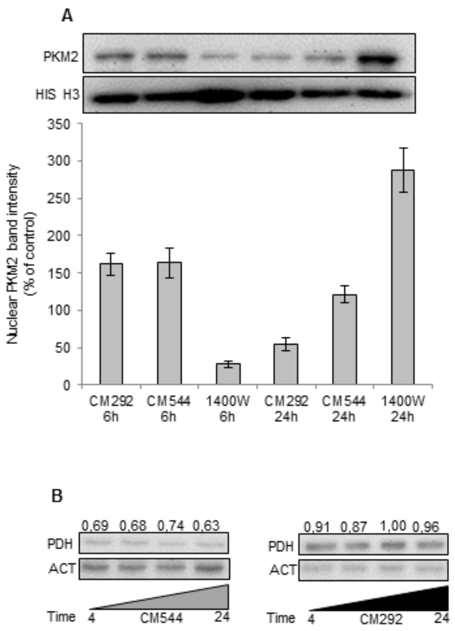

Figure S5. Supplemental to Fig. 5. BV2 microglial cells were treated with 200µM CM544, or 200µM CM292, or 10µM 1400W and used for: (A) Western blotting analysis of nuclear PKM2 at indicated time. Histone H3was used as loading control. The images are representative of one out of three separate experiments; (B) Time course of PDH. At each indicated time, cells were collected, and total extract analyzed by Western blotting. β–actin was used as loading control. The images are representative of one out of three separate experiments.

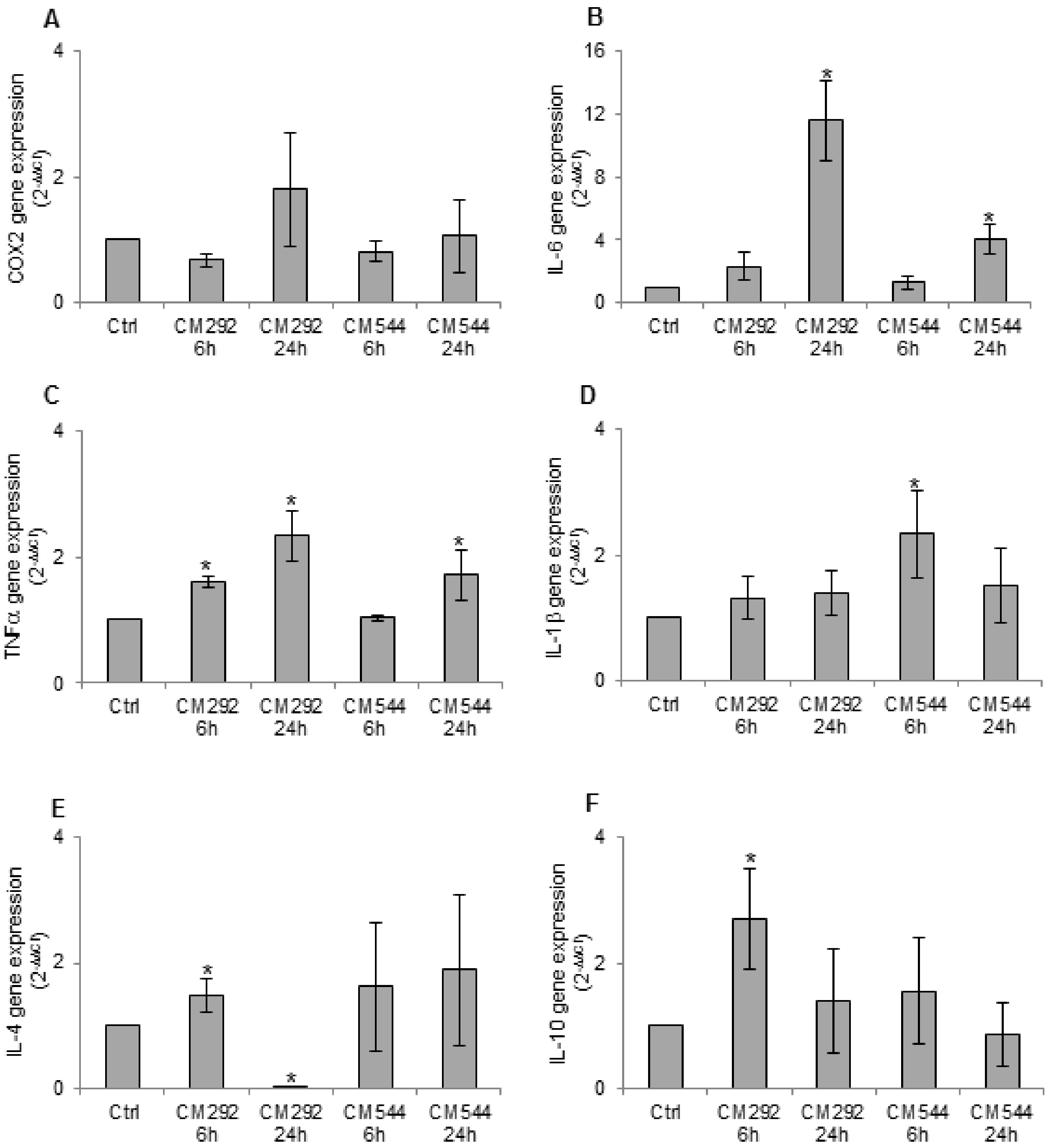

Figure S6. Supplemental to Fig. 6.BV2 microglial cellswere exposed to 200μM CM544 or 200μM CM292 for 6 and 24h, and sample used for qRT-PCR of the indicated genes. Gene expression values were normalized to Gapdh and presented as $2^{-\Delta\Delta Ct}$. Relative mRNA gene abundance in untreated cells was assumed to be 1 (control). Data represent mean ± SD (n = 3). *p < 0.05 vs. control cells.

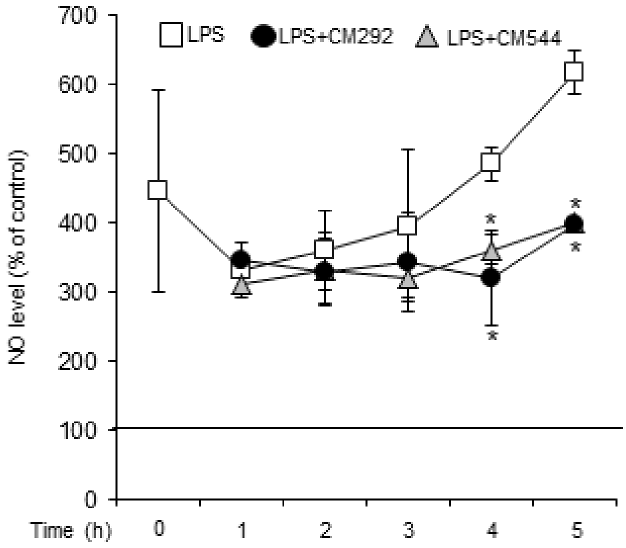

Figure S7. Supplemental to Fig. 6.BV2microglial cells, pre-treated for 18h with 10µg/mL LPS, were exposed for the indicated time to 200µM CM292 or 200µM CM544. NO production was detected by Griess reagent (absorbance of control cells was assumed as 100%). Data represent mean ± SD (n=3). *$p < 0.05$ vs LPS treated cells at the same time point.

**Compound**	**cLogD$_{7.4}$**	**cBCF**
**CM292**	**-4.51**	**-0.91**
**CM544**	**-4.67**	**-1.09**

Table S1. Calculated lipophilicity and bioconcentration factor of CM292 and CM544 by means of the program ACDlab v. 6.0
